# Golgi phosphoprotein 3 induces autophagy and epithelial–mesenchymal transition to promote metastasis in colon cancer

**DOI:** 10.1038/s41420-022-00864-2

**Published:** 2022-02-21

**Authors:** Li-Yun Gong, Ting Tu, Jing Zhu, Ao-Ping Hu, Jun-Wei Song, Jing-Qiang Huang, Yi Yang, Zeyao Zhu, Yu Chen

**Affiliations:** 1 Guangdong Provincial Key Laboratory for Genome Stability and Disease Prevention, Department of Biochemistry and Molecular Biology, School of Basic Medical Sciences, Health Science Center, Shenzhen University, 518060 Shenzhen, Guangdong P. R. China; 2 School of Dentistry, Shenzhen University Health Science Center, 518060 Shenzhen, Guangdong P. R. China; 3 Department of Anatomy, Histology and Developmental Biology, School of Basic Medical Sciences, Shenzhen University Health Science Centre, 518060 Shenzhen, Guangdong P. R. China; 4 School of Life Science, Southern University of Science and Technology, 518060 Shenzhen, Guangdong P. R. China; 5 Department of Gynecology, Shenzhen Second People’s Hospital/The First Affiliated Hospital of Shenzhen University Health Science Center, 3002 Sungang West Road, 518035 Shenzhen, Guangdong province P. R. China

**Keywords:** Prognostic markers, Metastasis

## Abstract

In this study, we aimed to investigate whether and how Golgi phosphoprotein 3 (GOLPH3) facilitates colon cancer metastasis via the regulation of autophagy and epithelial–mesenchymal transition (EMT). The role GOLPH3 plays in colon cancer metastasis was analyzed using western blotting, immunohistochemistry, transwell, wound-healing, and zebrafish assays. Autophagy and EMT were assessed via RNA-sequencing (RNA-seq) analysis, mRFP-GFP-LC3 reporter assays, and their related markers. Significant associations were found between colon cancer clinical and pathological stages and poor prognosis. GOLPH3 facilitates colon cancer metastasis, both in vitro and in vivo. RNA-seq analysis of GOLPH3-overexpressing and control cell models revealed that GOLPH3 enhances EMT and autophagy. Moreover, examination of autophagic, epithelial, and mesenchymal markers in GOLPH3-overexpressing, -silenced, and control cell lines revealed that GOLPH3 promotes EMT and autophagy. When autophagy was inhibited, GOLPH3-promoted metastasis and EMT were counteracted in vitro and in vivo. Using RNA-seq, PI3K/Akt signaling was identified as the key downstream pathway on which GOLPH3 acts. Mechanistically, we demonstrated that GOLPH3 stimulates autophagy and induces EMT via the suppression of the phosphorylation of protein kinase B (Akt) at Ser473. In summary, GOLPH3 induces autophagy and EMT, promoting metastasis in colon cancer. Beyond this, and in contrast to conventional perspectives, we discovered that GOLPH3 represses the phosphorylation of Akt at Ser473.

## Introduction

Colon cancer is one of the most common cancers worldwide (1,148,515 cases, 6.0% of the total) [1]. The survival rate declines from 90.6% to 72.2% and 14.7% for patients diagnosed with localized, regional, and distant stages, respectively [2]. A deeper understanding of the molecular mechanisms that affect colon cancer metastasis might improve the prognosis of colon cancer patients.

Epithelial–mesenchymal transition (EMT) is a cell remodeling process in which cells gradually lose their epithelial characteristics and shift into the mesenchymal state [3]. EMT stimulates tumor initiation, tumor progression and metastasis, and resistance to therapy [4, 5]. There is also compelling evidence to suggest that EMT facilitates colon cancer metastasis, with studies showing that EMT can be stimulated through signaling pathways such as Wnt/β-Catenin [6, 7], AKT/GSK 3β/Snail [8, 9], and PI3K/AKT [10–12]. The inhibition of such signaling represses EMT and tumor metastasis. However, more work is needed to identify the molecular networks that modulate colon cancer metastasis through the regulation of EMT.

During the process of EMT, a stable nutrient support system is required for cancer cells to survive independently [13]. Autophagy is a conserved intracellular process whereby cytoplasmic substances are engulfed by autophagosomes, which can later be degraded by fusing with lysosomes [14]. Autophagy allows large cargoes, such as protein aggregates, nucleic acids, organelles, and pathogens, as well as energy stores, including lipid membranes and glycogen, to be degraded and recycled, supporting cells to survive stress and promoting EMT and tumor metastasis [15, 16]. Cellular conditions that lead to abnormal ATP or cytokine levels and calcium fluctuation, including hypoxia and nutrient deprivation, induce the disruption of endoplasmic reticulum (ER) homeostasis and finally give rise to the accumulation and aggregation of unfolded proteins in the ER, also known as ER stress [17]. In response to ER stress, ER transmembrane proteins are stimulated, specifically inositol-requiring 1α (IRE1α), PKR-like ER kinase (PERK), and activating transcription factor 6α (ATF6α) [18]. IRE1α phosphorylates mitogen-activated protein kinase (MAPK), a stress-associated protein kinase that interacts with c-Jun N-terminal kinase (JNK) and mediates stress-induced autophagy [19, 20]. PERK mainly phosphorylates eukaryotic translation initiator factor-2α (eIF2α) and allows the selective translation of activating transcription factor 4 (ATF4) mRNA [21, 22]. ATF6, the least characterized branch, translocates to the Golgi apparatus and facilitates autophagy via the regulation of the expression of various genes related to ER-associated degradation of misfolded proteins [23, 24]. Notably, the above-mentioned three branches are linked with the phosphatidylinositol 3-kinase (PI3K)/Akt/mammalian target of rapamycin (mTOR) signaling, making it one of the most crucial regulators of autophagy [18, 25].

Evidence suggests that the Golgi apparatus plays a crucial role in tumorigenesis and cancer development [26, 27]. Golgi phosphoprotein 3 (GOLPH3), also known as GPP34, GMx33, and MIDAS, is a highly conserved 34 kDa protein located in the trans-Golgi network [28] and has a variety of functions. GOLPH3 promotes cell transformation and influences cell size and the cell cycle by enhancing the mTOR signaling pathway [29, 30]; it also regulates cell surface receptor trafficking via the inhibition of Rab5-mediated endocytosis and degradation of epidermal growth factor receptor (EGFR) and downstream activation of the PI3K/Akt/mTOR axis [31]. In response to DNA damage, DNA damage protein kinase (DNA-PK) phosphorylates GOLPH3, which enhances Golgi dispersal, resulting in the inhibition of cell apoptosis [32, 33]. GOLPH3 also facilitates Golgi extension and expedites vesicular trafficking, which drives malignant secretion via myosin-18A and PITPNC1 [34, 35]. Accumulating evidence suggests that GOLPH3 is associated with cancer-related phenotypes and poor prognosis in various solid tumors, including colon cancer [36]. Given the role of GOLPH3 in vesicular secretion and its close interaction with the PI3K/Akt/mTOR pathway, we hypothesized that GOLPH3 may promote EMT by regulating autophagy. In this work, we demonstrate the role GOLPH3 plays in colon cancer metastasis via the regulation of autophagy and EMT.

## Results

### GOLPH3 overexpression is significantly associated with colon cancer stage, tumor–node–metastasis (TNM) classification, and poor prognosis

**Fig. 1:**
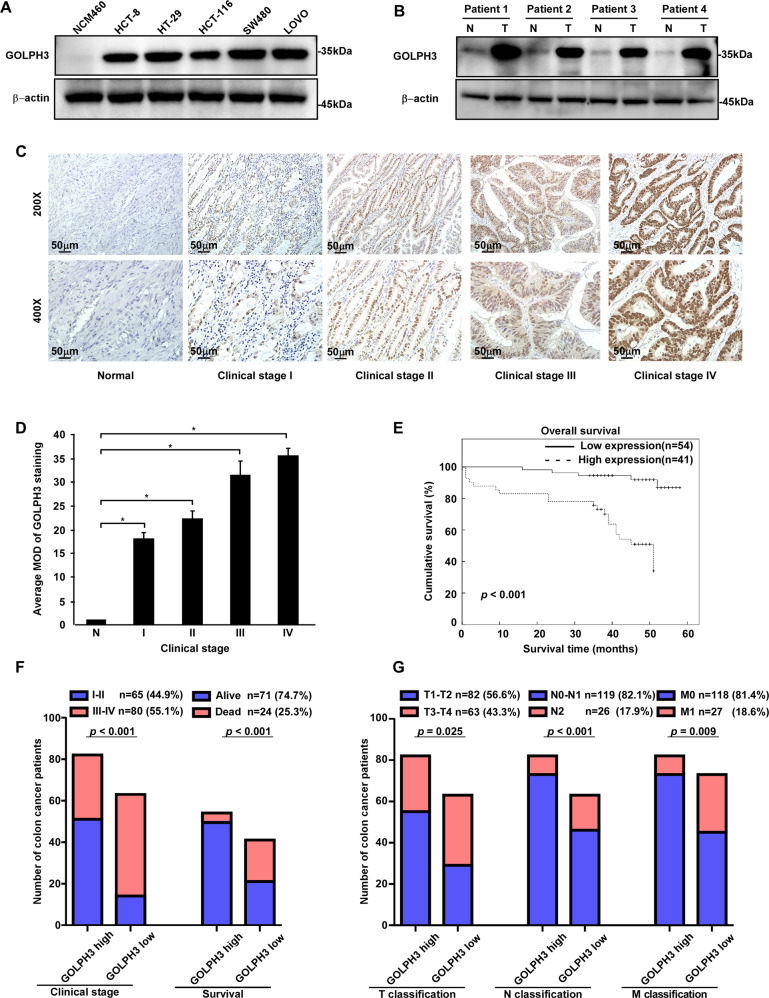
GOLPH3 overexpression is significantly associated with colon cancer clinical-stage, TNM classification, and poor prognosis. **A** Western blotting analysis of GOLPH3 levels in colon cancer cell lines, including HCT-8, HT-29, HCT-116, SW480, and LOVO, and in the normal human colon epithelial cell line NCM460. β-actin served as the internal reference. Compared with the expression levels in NCM460 cells, colon cancer cell lines expressed high levels of GOLPH3. **B** Western blotting analysis of GOLPH3 levels in colon cancer tissues and paired non-cancerous adjacent tissue. GOLPH3 was upregulated in the four colon cancer tissues. **C** IHC staining of GOLPH3 in colon cancer tissues at different clinical stages. **D** Quantitative analysis of the data shown in **C**. GOLPH3 levels were positively correlated with clinical-stage. **E** Overall survival was analyzed using Kaplan–Meier analysis and the log-rank test; the level of GOLPH3 was negatively correlated with overall survival (*P* < 0.001). **F, G** Chi-square analysis: the differences in GOLPH3 levels between different clinical stages (*P* < 0.001) and T classification (*P* = 0.025), N classification (*P* < 0.001), and M classification (*P* = 0.009).

To evaluate the association between GOLPH3 and colon cancer, we investigated GOLPH3 expression in colon cancer cell lines and tissues. Western blotting revealed that GOLPH3 was highly expressed in colon cancer cell lines (HCT-8, HT-29, HCT-116, SW480, and LOVO), but barely expressed in normal human colon epithelial cells (NCM460) (Fig. 1A). GOLPH3 protein levels were consistently and significantly upregulated in four colon cancer tissues compared with the levels in matched, adjacent non-cancerous tissues (Fig. 1B). Quantitative analyses of immunohistochemistry (IHC) staining indicated that GOLPH3 levels increased in colon cancer tissue as the clinical-stage advanced and were elevated overall compared with the levels in normal tissue (Fig. 1C, D). Kaplan–Meier analysis and log-rank tests revealed that GOLPH3 levels were negatively correlated with overall survival (*P* < 0.001; Fig. 1E). Chi-squared tests revealed a positive relationship between GOLPH3 levels and clinical-stage (*P* < 0.001), cancer death (Fig. 1F, Tables 1, 2), T classification (*P* = 0.025), N classification (*P* < 0.001), and M classification (*P* = 0.009) (Fig. 1G, Tables 1, 2). Spearman rank analysis confirmed that there was a significant correlation between increasing GOLPH3 levels and progression in TNM stage (Table 3). The overall survival of patients with high and low GOLPH3 levels was compared within subgroups of clinical-stage, T classification, lymph node metastasis, and distant metastasis, which revealed a negative correlation between patient prognosis and GOLPH3 levels (Supplemental Fig. S1). Furthermore, multivariate survival analysis showed that GOLPH3 expression was an independent prognostic factor for evaluating patient outcomes (Table 4). Taken together, these findings showed that GOLPH3 expression levels were correlated with the degree of malignant features in colon cancer.

**Table 1:** Clinicopathologic characteristics of patient samples and expression Of GOLPH3 in colorectal cancer.

Clinicopathological factors	No. (%)
Gender	
Male	80 (55.2)
Female	65 (44.8)
Age (years)	
≤65	70 (48.3)
>65	75 (51.7)
Grade(AJCC)	
I	32 (22.1)
II	33 (22.8)
III	53 (36.6)
IV	27 (18.6)
T classification	
T_1_	32 (22.1)
T_2_	50 (34.5)
T_3_	32 (22.1)
T_4_	31 (21.4)
N classification	
N_0_	72 (49.7)
N_1_	47 (32.4)
N_2_	26 (17.9)
M classification	
M_0_	118 (81.4)
M_1_	27 (18.6)
Survival (*n* = 95)	
Alive	71 (74.7)
Dead	24 (25.3)
Survial time of low expression	
Mean	46.69
Median	49.00
Survival time of high expression	
Mean	34.83
Median	39.00
Expression of GOLPH3	
Low expression	82 (56.6)
High expression	63 (43.4)

**Table 2:** Correlation between the clinical pathologic features and expressions of GOLPH3.

Characteristics	*GOLPH3*		*P-*value
	Low or none	High	
Gender			0.401
Male	48	32	
Female	34	31	
Age (yrs)			0.093
≤65	45	25	
>65	37	38	
Clinical stage (AJCC Grade)			<0.001
I	25	7	
II	26	7	
III	21	31	
IV	10	18	
T classification			0.025
T1	21	11	
T2	34	18	
T3	17	14	
T4	10	20	
N classification			<0.001
N0	54	18	
N1	19	28	
N2	9	17	
M classification			0.009
M0	73	45	
M1	9	18	
Survival (*n* = 95)			<0.001
Alive	50	21	
Dead	4	20

**Table 3:** Spearman correlation analysis between GOLPH3 expression and clinical pathologic factors.

Variables	GOLPH3 expression level	
	Spearman correlation	*P-*value
Gender	0.077	0.356
Age	−0.151	0.070
Clinical stage	0.368	<0.001
T classification	0.223	0.007
N classification	0.363	<0.001
M classification	0.224	0.007
Survival	−0.383	<0.001

**Table 4:** Univariate and multivariate analysis of different prognostic parameters in patients with colorectal cancer.

Univariate analysis			Multivariate analysis		
	*P*	Regression coefficient (SE)	*P*	Relative risk	95% confidence interval
Clinical stage	0.031	1.592 (0.216)	0.048	0.417	0.175–0.994
Expression of GOLPH3	<0.001	8.648 (0.558)	0.002	6.539	2.036–21.000
T classification	0.030	1.496 (0.185)	0.042	1.499	1.015–2.213
N classification	0.005	2.192 (0.277)	0.032	2.429	1.077–5.477
M classification	0.021	2.732 (0.434)	0.033	4.886	1.140–20.943
Age	0.033	0.382 (0.451)	0.057	0.407	0.161–1.028

### GOLPH3 overexpression promotes colon cancer migration and invasion in vitro

**Fig. 2:**
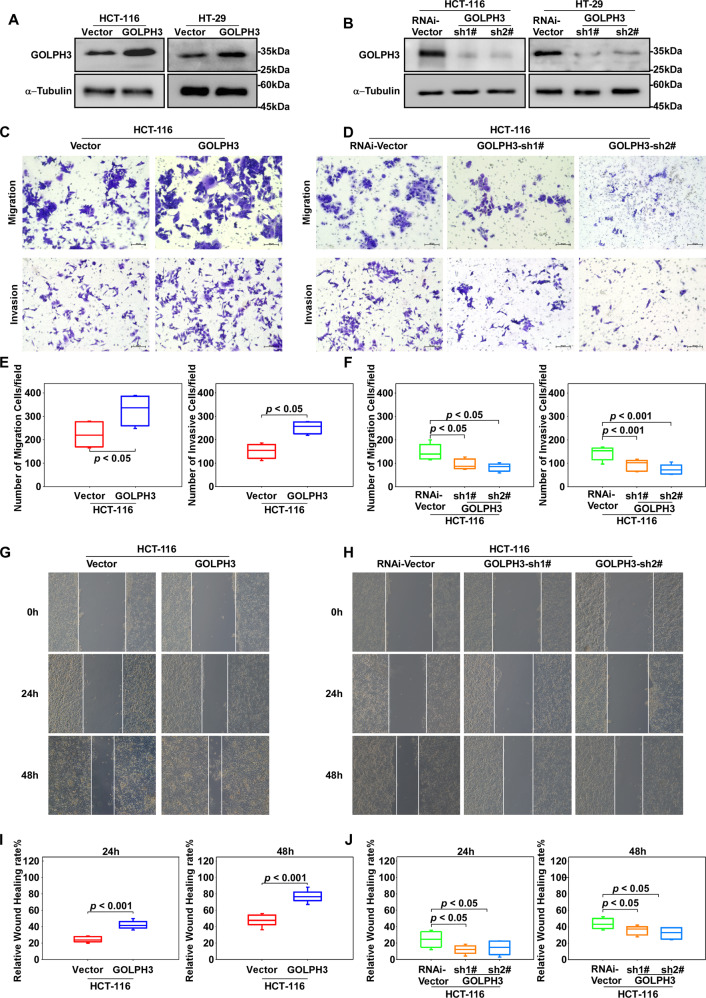
GOLPH3 overexpression promotes colon cancer migration and invasion in vitro. **A** Western blotting analysis of GOLPH3 levels in HCT-116 and HT-29 cell lines transfected with PMSCV-Vector and PMSCV-GOLPH3. *GOLPH3*-overexpressing cell lines and their controls were successfully constructed. **B** Western blotting analysis of GOLPH3 levels in HCT-116 and HT-29 cell lines transfected with pSuper-Vector or pSuper-GOLPH3-sh1#/sh2#. **C**, **D** Transwell assays were used to analyze the migration and invasion ability in HCT-116-PMSCV-Vector, HCT-116-PMSCV-Mcherry-GOLPH3, HCT-116-pSuper-Vector, and HCT-116-pSuper-GOLPH3-sh1#/sh2# cells. **E**, **F** Quantitative analysis of the data shown in **C**, **D**; the overexpression of *GOLPH3* enhanced migration (*P* < 0.05) and invasion (*P* < 0.05) ability; silencing of *GOLPH3* inhibited migration (*P* < 0.05) and invasion (*P* < 0.001) ability. **G**, **H** The migration ability of HCT-116-PMSCV-Vector, HCT-116-PMSCV-GOLPH3, HCT-116-pSuper-Vector, and HCT-116-pSuper-GOLPH3-sh1#/2# were evaluated using a wound-healing assay. **I**, **J** Quantitative analysis of the data shown in **G**, **H**; the overexpression of *GOLPH3* enhanced migration (*P* < 0.001), whereas silencing of *GOLPH3* inhibited migration (*P* < 0.05).

To further explore the role of GOLPH3 in colon cancer progression, we transfected HCT-116 and HT-29 cells with PMSCV-Vector, PMSCV-Vector-GOLPH3, pSuper-Vector, and pSuper-GOLPH3-sh1#/sh2# lentivirus, respectively, to construct stable GOLPH3-overexpression and -silenced cell lines (Fig. 2A, B). Transwell assays demonstrated that compared with the corresponding controls, GOLPH3 overexpression improved migration and invasion (Fig. 2C, E), while GOLPH3 silencing suppressed cells’ metastatic behavior (Fig. 2D, F). Wound-healing assays revealed that GOLPH3 markedly promoted HCT-116 cell migration (Fig. 2G, I). In contrast, GOLPH3 downregulation inhibited HCT-116 cell migration (Figs. 2H, J). Collectively, these findings showed that GOLPH3 enhanced migration and invasion in colon cancer.

### Upregulation of GOLPH3 promotes colon cancer metastasis in vivo

**Fig. 3:**
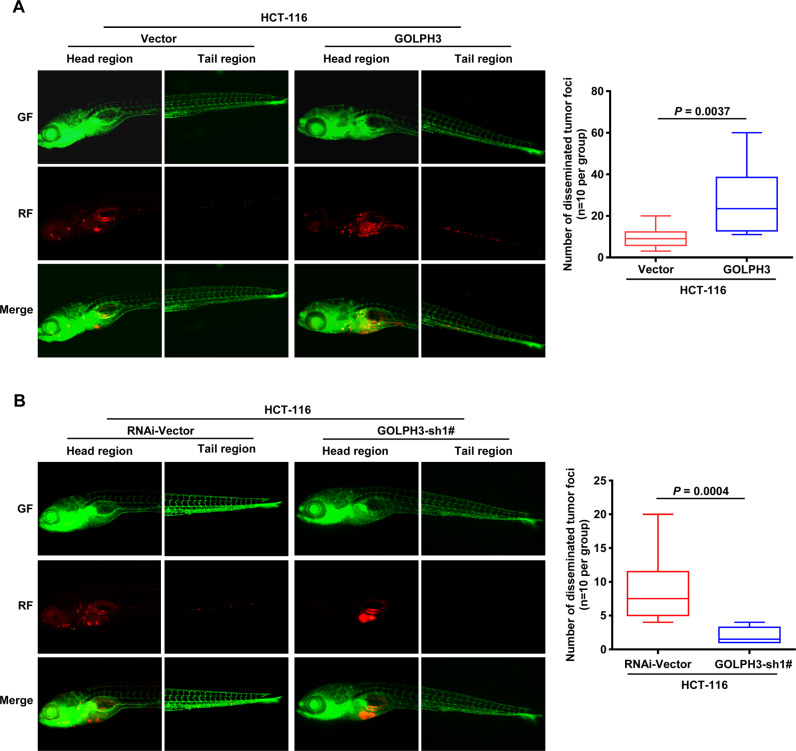
GOLPH3 overexpression induces metastasis in colon cancer in vivo. **A** Zebrafish models injected with HCT-116-PMSCV-Vector and HCT-116-PMSCV-GOLPH3 cells were used. Green fluorescence indicates zebrafish, and red fluorescence was used to mark the cell lines. At 1-week post-injection, the disseminated tumor foci count was significantly higher in the *GOLPH3*-overexpression model (*P* = 0.0037). **B** HCT-116-pSuper-Vector and HCT-116-pSuper-GOLPH3 sh1# cells were also used to establish a zebrafish model. In contrast to **A**, the disseminated tumor foci count was significantly lower in the *GOLPH3*-silencing model (*P* = 0.0004).

We established an in vivo model by separately microinjecting HCT-116-PMSCV-Vector, HCT-116-PMSCV-GOLPH3, HCT-116-pSuper-Vector, and HCT-116-pSuper-GOLPH3 sh1# cells into the yolk sacs of zebrafish embryos. Within a week post-injection, increased numbers of disseminated tumor foci were observed in the zebrafish tail region in the GOLPH3-overexpression group compared with the numbers seen in the corresponding control group (Fig. 3A). The opposite phenomenon was observed in the GOLPH3-silenced group (Fig. 3B). These results provided strong evidence that GOLPH3 increased metastasis of human colon cancer cells in vivo.

### GOLPH3 overexpression induces colon cancer metastasis by enhancing EMT

**Fig. 4:**
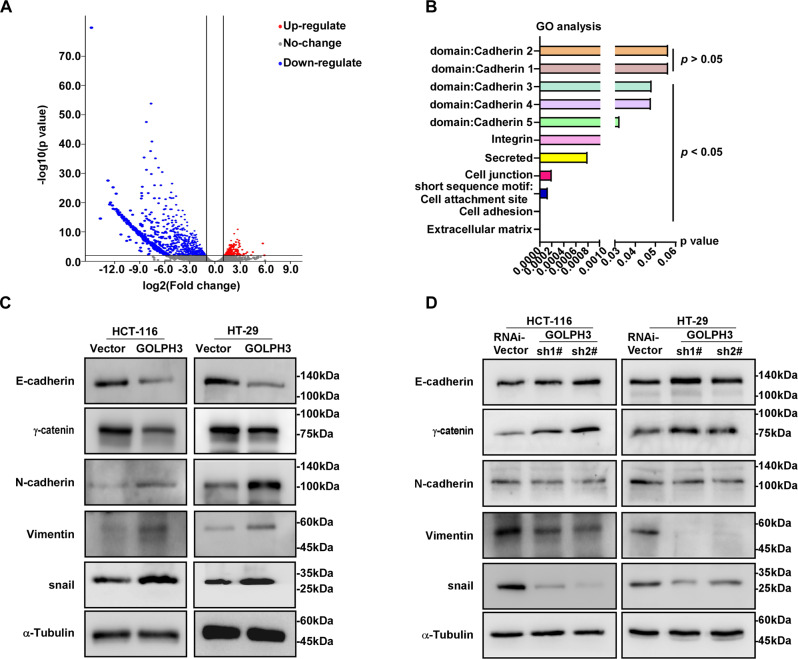
GOLPH3 overexpression promotes colon cancer metastasis by enhancing EMT. **A** A volcano plot showing RNA-seq-detected gene expression in HCT-116-PMSCE-Vector and HCT-116-PMSCV-GOLPH3 cells. Differentially expressed genes were filtered using a *P*-value < 0.05 as a threshold. Red spots represent upregulated genes, green spots represent downregulated genes, and blue spots represent genes that showed no obvious change. **B** Differentially expressed genes between HCT-116-PMSCV-Vector and HCT-116-PMSCV-GOLPH3 cells assessed using GO analysis. **C** Western blotting analysis of EMT-related markers in *GOLPH3*-overexpressing cells and their controls; α-Tubulin was used as an internal reference. In *GOLPH3*-overexpressing cells, epithelial markers (E-cadherin, γ-catenin) were downregulated, while mesenchymal markers (N-cadherin, vimentin, and Snail) were upregulated. **D** The opposite results to those obtained in **C** were observed when *GOLPH3* was silenced.

RNA sequencing (RNA-seq) was used to investigate gene expression in HCT-116-PMSCV-Vector and HCT-116-PMSCV-GOLPH3 cells, while Gene Ontology (GO) was used to annotate the various functional genes. By comparing the differentially expressed genes (Fig. 4A), we noticed that GOLPH3 was associated with EMT (Fig. 4B). EMT is a crucial event during tumor metastasis. Therefore, we explored the impact of GOLPH3 on EMT. We investigated the levels of EMT-related markers in GOLPH3-overexpressing and -silenced colon cancer cell lines. Levels of E-cadherin and γ-catenin, which are classical epithelial markers, were downregulated in GOLPH3-overexpressing cells. Meanwhile, levels of the mesenchymal markers N-cadherin, vimentin, and Snail were upregulated (Fig. 4C, Supplemental Fig. S2). In contrast, in GOLPH3-silenced cells, epithelial markers were upregulated, and mesenchymal markers were downregulated (Fig. 4D, Supplemental Fig. S2). These results suggested that GOLPH3 promotes colon cancer metastasis by enhancing EMT.

### GOLPH3 overexpression enhances autophagy in colon cancer

**Fig. 5:**
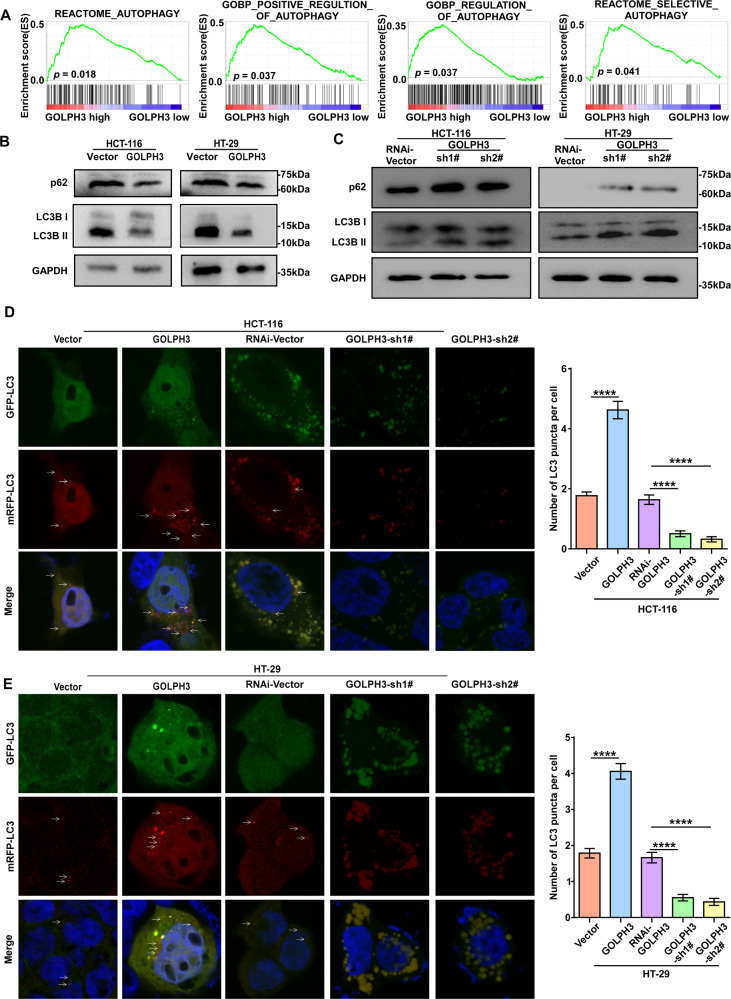
GOLPH3 overexpression increases autophagy in colon cancer. **A** GSEA analysis of the correlation between RNA-seq-detected GOLPH3 gene expression and cell autophagy genes. **B** Western blotting analysis of autophagy markers in *GOLPH3*-overexpressing cells and their controls; GAPDH was used as an internal reference. The levels of autophagy inhibition markers (p62 and LC3 II) decreased in *GOLPH3*-overexpressing cells. **C** p62 and LC3 II accumulated when *GOLPH3* was silenced. **D** Fluorescence microscopy observation of HCT-116 cells transfected with mRFP-GFP-LC3 plasmid. **E** A similar phenomenon to that seen in **D** was observed in HT-29 cells.

Gene set enrichment analysis (GSEA) analysis of the RNA-seq results based on the GSE77953 dataset was performed to investigate the biological functions of HCT-116-PMSCV-Vector and HCT-116-PMSCV-GOLPH3 cells. The findings suggested that GOLPH3 expression was positively associated with colon cancer cell autophagy (Fig. 5A). The extent of autophagy was evaluated by analyzing the levels of p62 and microtubule-associated protein 1 A/1B-light chain 3 (LC3), including a cytosolic form of LC3 (LC3-I), and LC3-phosphatidylethanolamine conjugate (LC3 II). As shown in Fig. 5B, p62 and LC3 II levels were downregulated in GOLPH3-overexpressing cells, while the opposite effect was observed in GOLPH3-silenced cell lines (Fig. 5C). However, LC3 I levels remained constant in GOLPH3-overexpressing and -silenced cells compared with those in their paired controls, indicating that GOLPH3 interferes with autophagy after autophagosomes have formed. Autophagy flux was visualized using RFP-GFP-targeted LC3 fluorescence. We observed that GOLPH3 overexpression increased the level of autolysosomes (red dots), while GOLPH3 silencing increased the number of autophagosomes (yellow dots) in HCT-116 cells (Fig. 5D) and HT-29 cells (Fig. 5E). These results further demonstrated that GOLPH3 promotes autophagy by stimulating the fusion of autophagosomes and lysosomes.

### Upregulation of GOLPH3 promotes EMT in colon cancer cells by inducing autophagy, both in vitro and in vivo

**Fig. 6:**
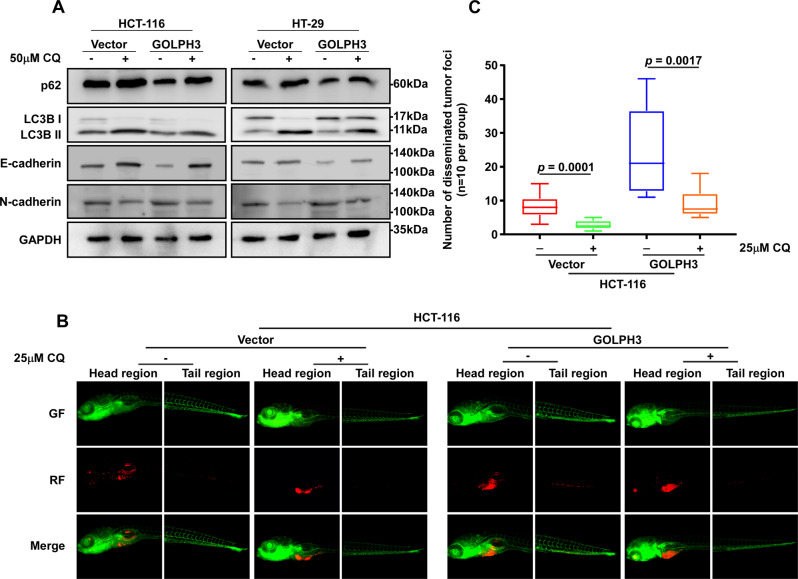
GOLPH3 overexpression enhances EMT of colon cancer cells by inducing autophagy, both in vitro and in vivo. **A** *GOLPH3*-overexpressing and control HCT-116 and HT-29 cells were cultured in regular cell culture solution with or without 50 μM CQ. Western blotting analysis of autophagy inhibition markers (p62 and LC3 II), epithelial markers (E-cadherin), and mesenchymal markers (N-cadherin) was performed. The overexpression of *GOLPH3* induced downregulation of p62, LC3 II, and E-cadherin, while it induced upregulation of N-cadherin. When autophagy was suppressed by CQ, E-cadherin levels increased, and N-cadherin levels decreased in each group. **B** *GOLPH3*-overexpressing HCT-116 cells and their controls and *GOLPH3-*silenced HCT-116 cells and their controls were injected into zebrafish and then cultured in medium solution with or without an autophagy suppressor (25 μM CQ). **C** A decreased quantity of disseminated tumor foci was observed in the *GOLPH3*-overexpression model (*P* = 0.0001) and its control (*P* = 0.0017).

We verified the hypothesis that the upregulation of GOLPH3 promotes EMT in colon cancer cells by inducing autophagy, both in vivo and vitro, using chloroquine (CQ), an autophagy inhibitor that blocks the fusion of autophagosomes with lysosomes [37, 38]. HCT-116-PMSCV-Vector (control), HCT-116-PMSCV-Vector-GOLPH3 (GOLPH3 overexpression), HT-29-PMSCV-Vector (control), and HT-29-PMSCV-Vector-GOLPH3 (GOLPH3 overexpression) cells were cultured in regular cell culture solution with or without 50 μM CQ. In the regular culture solution group, p62 and LC3 II levels were repressed in GOLPH3-overexpressing cells compared with their levels in the controls. By contrast, EMT markers were upregulated in GOLPH3-overexpressing cells. Western blotting analysis of p62 and LC3 II confirmed that, when cultured in CQ, the inhibition of autophagy was observed in GOLPH3-overexpressing and control cells. And, interestingly, EMT markers were downregulated in both cells (Fig. 6A). In addition, zebrafish models microinjected with HCT-116-PMSCV-Vector (control) or HCT-116-PMSCV-Vector-GOLPH3 (GOLPH3 overexpression) in the head region were established. Both zebrafish models were cultured in medium with or without 25 μM CQ. At 1 week after injection, in the regular culture medium group, the number of disseminated tumor foci was elevated in the zebrafish tail region in the GOLPH3-overexpressing model compared with the number in the corresponding control. In the 25 μM CQ-cultured group, the number of disseminated tumor foci was decreased in both the GOLPH3-overexpression and control models (Figs. 6B, 6C). These results further illustrated that GOLPH3 elicits EMT via boosting autophagy; by suppressing autophagy using chloroquine, the effect of GOLPH3 on EMT was counteracted.

### GOLPH3 facilitates autophagy via suppressing the phosphorylation of Akt at Ser473

**Fig. 7:**
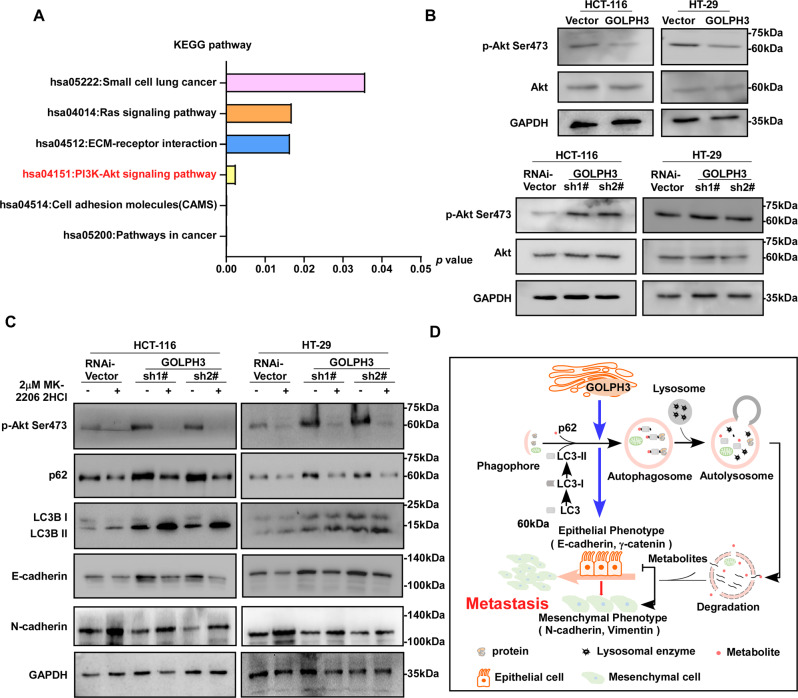
GOLPH3 facilitates autophagy via the suppression of phosphorylation of Akt at Ser473. **A** KEGG (Kyoto Encyclopedia of Genes and Genomes) analyses differentially expressed genes between the HCT-116-PMSCE-Vector and HCT-116-PMSCV-GOLPH3 cells. **B** Western blot analysis of GOLPH3-overexpressing and control HCT-116 and HT-29 cell lines. Akt Ser473 phosphorylation was decreased in GOLPH3-overexpressing cells. In GOLPH3-silenced colorectal cancer cells, Akt Ser473 phosphorylation was enhanced. **C** MK-2206 2HCl was used to repress Akt phosphorylation. P62 and E-cadherin were stimulated, and N-cadherin was repressed when GOLPH3 was silenced. When CRC cell lines were cultured in Akt Ser473 phosphorylation inhibitor, P62 and E-cadherin were repressed, and N-cadherin was enhanced. GAPDH was used as an internal reference. **D** The above results demonstrated that GOLPH3 induces autophagy and EMT to promote metastasis in colon cancer, via the repression of Akt Ser473 phosphorylation.

Signal pathway enrichment was next analyzed based on the differential expression of genes between HCT-116-PMSCV-Vector and HCT-116-PMSCV-GOLPH3 cells, as examined by RNA-seq. Notably, GOLPH3 has correlated with the PI3K/Akt signaling pathway (Fig. 7A). Western blotting confirmed that compared with the level in paired controls, the level of Akt phosphorylated at Ser473 was decreased in GOLPH3-overexpressing colon cancer cells, while the opposite effect was observed in GOLPH3-silenced colon cancer cells (Fig. 7B). As shown in Fig. 7C, compared with what was observed in the controls, GOLPH3 silencing resulted in enhanced Akt Ser473 phosphorylation and downregulation of autophagy and EMT. This suggested that GOLPH3 regulates autophagy by suppressing Akt Ser473 phosphorylation. We next added MK-2206 2HCl to the culture solution to inhibit Akt phosphorylation, and the inhibitory effect was confirmed using western blotting. Compared with that seen in colon cancer cells cultured in the regular culture solution, the inhibition of autophagy and EMT by GOLPH3 RNAi was counteracted when Akt Ser473 phosphorylation was repressed by MK-2206 2HCl. As shown in Fig. 7C, colon cancer cells cultured with the Akt phosphorylation inhibitor exhibited upregulation of autophagy (a decrease in the autophagy inhibition marker p62). EMT was also promoted (with a decrease in epithelial markers and an increase in mesenchymal markers) by Akt inhibition. In summary, our results showed that GOLPH3 inhibits the phosphorylation of Akt to facilitate autophagy, which enhances EMT and promotes metastasis in colon cancer (Fig. 7D).

## Discussion

The Golgi apparatus, a eukaryotic organelle, is essential for macromolecule formation and secretion. In recent years, the involvement of the Golgi apparatus in cancer has been revealed. GOLPH3 was identified as the first Golgi resident oncoprotein [28]; since then, compelling evidence has shown that GOLPH3 accelerates cancer progression. It has been confirmed that GOLPH3 can exacerbate cancer progression via various pathways: (1) GOLPH3 binds to and acts as a downstream effector of phosphatidylinositols (PIs), notably PI 4-phosphate (PI4P), leading to the enlargement of the Golgi apparatus, as well as vesicular secretion; (2) GOLPH3 activates mTOR signaling by phosphorylating S6 kinase (S6K) and Akt, resulting in changes in cell size and cell growth; (3) GOLPH3 mediates intra-Golgi trafficking of protein glycosyltransferases and increases the glycosylation of cancer-relevant glycoproteins; and (4) GOLPH3 interacts with DNA damage protein kinase (DNA-PK) to create a tensile force that causes Golgi dispersal, which maintains cell survival after DNA damage [36, 39].

It has been shown that GOLPH3 is associated with the development of colon cancer and accounts for chemotherapy resistance [40–43]. In agreement with previous research, our results revealed that GOLPH3 expression was positively correlated with cancer progression and indicated a poor prognosis for patients with colon cancer. Our results also suggested that GOLPH3 promotes colon cancer metastasis. In the present study, we established GOLPH3-overexpression and -silenced colon cancer cell lines and their corresponding controls; we also established zebrafish models. Using these cell lines and models, we confirmed that GOLPH3 promotes cancer cell invasion and migration, both in vitro and in vivo.

EMT is a process during which epithelial cells lose their junctions and apical–basal polarity and are reprogrammed into a mesenchymal phenotype. EMT expedites the stem-like behavior of individual cells and facilitates the malignant features of cancer cells [44, 45]. EMT plays a crucial role in tumor initiation and progression, and the extent of EMT is associated with metastatic potential [3]. Thus, we proposed that GOLPH3 promotes colon cancer progression by enhancing EMT and confirmed this hypothesis via further experiments. EMT-associated transcription factors (TFs), such as Snail, Zeb1, and Twist, play a central role in inducing EMT [46, 47]. TFs repress epithelial genes such as E-cadherin and activate mesenchymal genes such as N-cadherin [46, 48]. Hence, a distinct change that can be easily detected during this process is the downregulation of the expression of epithelial markers, such as E-cadherin and γ-catenin, and the acquisition of mesenchymal markers, such as vimentin, N-cadherin, and Snail [49–51]. Therefore, we used these markers in this study.

During EMT, a stable nutrient support system is required for cancer cell transition and survival. Autophagy, a stress-induced self-degradation process, has been shown to be a reliable way for cancer cells to achieve intracellular self-renewal and self-sufficiency. Autophagy enables cancer cells to survive in adverse environments, making it a stimulating and supportive mechanism for EMT [52–54]. Studies have confirmed that PI3K/Akt/mTOR signaling is one of the most crucial regulators of autophagy [18, 25]. Given that GOLPH3 facilitates vesicular secretion and has a close interaction with the PI3K/Akt/mTOR pathway, we hypothesized that GOLPH3 may promote EMT by regulating autophagy.

During the process of autophagy, autophagosomes engulf cytoplasmic components. p62 is an autophagy adapter protein that binds to ubiquitylated protein aggregates and delivers them to autophagosomes. The level of p62 in cells is inversely proportional to the extent of autophagy. The LC3 family is a group of byproducts resulting from autophagy and is considered to be a marker of autophagy. The cytosolic form of LC3, LC3 I, can be conjugated with phosphatidylethanolamine to form LC3 II, which is located in autophagosome membranes. After autophagosomes fuse with lysosomes, LC3 II will be degraded [55]. Taking these findings together, we chose p62 and LC3 as biomarkers to evaluate autophagy. We found that p62 and LC3 II levels were downregulated in GOLPH3-overexpressing colon cancer cell lines, while LC3 I level remained constant in both GOLPH3-overexpressing and -silenced cells compared with the level in their paired controls. This implied that GOLPH3 enhances autophagy after autophagosomes have formed. Furthermore, when Akt phosphorylation was inhibited by using MK-2206, the levels of p62 were downregulated, indicating enhanced autophagy; however, LC3 II levels were upregulated, suggesting that MK-2206 interferes in another step of the autophagy process.

Recently, Akt was identified to be a key component in regulating autophagy, while the Akt inhibitor MK-2206 was shown to be capable of inducing autophagy [56, 57]. Given the role of GOLPH3 in promoting autophagy, we hypothesized that GOLPH3 suppresses Akt, which contradicts the conventional conception that GOLPH3 promotes Akt [29]. Interestingly, we confirmed our hypothesis and showed that GOLPH3 inhibited Akt phosphorylation at Ser473; this contradicts the majority of previous studies, which reported that GOLPH3 activates the Akt/mTOR signaling to accelerate cancer progression [40, 58, 59]. Previous studies have confirmed that mTOR is a major suppressive regulator of the autophagic process and is regulated by starvation, growth factors, and cellular stresses [60]. By repressing Akt, which is an upstream regulator of mTOR, autophagy is thought to be upregulated, as demonstrated in our study. This correlation between GOLPH3 and Akt/mTOR has also been confirmed in ischemic models. In these models, GOLPH3 can act as both a sensor of Golgi stress and an initiator that triggers and propagates Golgi stress signals to downstream effectors [61]. When Golgi apparatus stress is ameliorated, GOLPH3 expression decreases, and the phosphorylation of the PI3K/Akt/mTOR pathway is promoted [62]. Previous studies that found GOLPH3 promotes Akt in colon cancer were performed using the cell lines HCT-116 [40], LoVo [58], and SW620 [63]. To the best of our knowledge, no study has constructed and used GOLPH3-overexpressing and -silenced cell lines to investigate the impact of GOLPH3 on Akt in HCT-116 and HT-29 cells. In addition, most of the studies that proved a promotive role of GOLPH3 on Akt focused on cancer proliferation rather than migration, invasion, and EMT. The above reasons might partially explain the contradiction; however, further research is needed to confirm this in different types of cancer.

Overall, we have demonstrated that GOLPH3 induces autophagy and EMT to promote metastasis in colon cancer via the repression of phosphorylation of Akt at Ser473. Further studies will provide insights into the therapeutic value of targeting GOLPH3 to control the progression of colon cancer.

## Materials and methods

### Patients and tissue specimens

This study was performed using four pairs of matched colon cancer tissues, non-cancerous tissues adjacent to the malignant lesion, and 145 paraffin-embedded colon cancer samples that were diagnosed histopathologically and clinically at The Affiliated Hospital of Shenzhen University between 2008 and 2013. For the clinical materials used in this study, prior patient consent and approval from the Institutional Research Ethics Committee (IREC) were obtained. Clinical and clinicopathological classification and staging were determined according to the American Joint Committee on Cancer (AJCC) criteria. Table 1 shows the clinical information for the samples. Tumor purity (%) in sections adjacent to the regions used for RNA extraction was estimated during routine histopathological analysis.

### Cell lines, culture conditions, and reagents

Human colon cancer cell lines (HCT-8, HT-29, HCT-116, SW480, and LOVO) were purchased from the American Type Culture Collection (ATCC, Manassas, VA, USA) and cultured in Dulbecco’s modified Eagle’s medium (DMEM) (HyClone, Logan, UT, USA) supplemented with 10% fetal bovine serum (FBS) (Gibco, Grand Island, NY, USA). Human normal colon mucosa cells (NCM460) were obtained from Dr. Ying Ying (Shenzhen University, Shenzhen, China). The Akt inhibitor MK-2206 2HCl was purchased from Selleck (S107811; 5 mg; Houston, TX, USA.) and dissolved in dimethyl sulfoxide (DMSO). The autophagy inhibitor, chloroquine diphosphate salt (CQ), was purchased from Sigma–Aldrich (C6628; 25 g; St. Louis, MO, USA) and dissolved in double-distilled water; all solutions were filtered through a 0.2 μm membrane and stored at 20 °C.

### Plasmids, retroviral infection, and transfection

The human GOLPH3 cDNA was amplified by PCR and cloned into PMSCV-Vector. To establish GOLPH3-overexpressing HCT-116 (HCT-116-PMSCV-Vector-GOLPH3) and HT-29 (HCT29-PMSCV-Vector-GOLPH3) cells, these cells were infected with lentiviral constructs expressing GOLPH3, together with their corresponding stable control cell lines (HCT-116-PMSCV-Vector and HT-29-PMSCV-Vector). Subsequently, two GOLPH3-silenced (pSuper-GOLPH3-sh1#, pSuper-GOLPH3-sh2#) and their corresponding stable control (pSuper-Vector) cell lines were obtained. The short hairpin RNAs (shRNAs) used in GOLPH3 knockdown were RNAi#1: GCATGTTAAGGAAACTCAGCC; RNAi#2: GCAGCGCCTCATCAAGAAAGT. GOLPH3 overexpression and silenced cell lines were selected for 7 days using 0.5 mg/mL puromycin (P8230; Solarbio, Beijing, China).

### Western blotting analysis

The colon cancer cells were lysed using cooled radioimmunoprecipitation assay (RIPA) lysis buffer. Proteins were extracted following a standard protocol, and their concentration was measured using a bicinchoninic acid (BCA) protein assay kit (Thermo Fisher Scientific, Waltham, MA, USA; #TK269550). Fresh tissue samples were ground to powder in liquid nitrogen and lysed using SDS-PAGE sample buffer. Protein samples (20 µg) were separated on 10% SDS-polyacrylamide gels and transferred to 0.45 µm PVDF membranes (Immobilon P, Millipore, Bedford, MA, USA). The membranes were blocked with 5% skim milk in Tris-buffered saline containing 0.1% Tween-20 (TBST) for 1 h at room temperature. The membranes were incubated overnight at 4 °C with antibodies recognizing the following proteins: β-actin (Sigma–Aldrich; #A5441;1:5000), mouse anti-GAPDH (1:5000, 200306-7E4, Zen BioScience Inc., Research Triangle Park, NC, USA), light chain 3B (LC3B) (Sigma–Aldrich; #L7543;1:1000), p62 (Abcam, Cambridge, MA, USA; #ab109012; 1:10000), Akt (Cell Signaling Technology (CST), Danvers, MA, USA; #4691 T; 1:1000), phospho-Akt Ser473 (CST; #4060 T; 1:2000), N-cadherin (BD Biosciences, San Jose, CA, USA; #610920; 1:1000), E-cadherin (BD Biosciences; #610181; 1:1000), Vimentin (CST; #5741 S; 1:1000), Snail (Abclonal, Wuhan, China; #A11794; 1:1000), γ-catenin (CST; #2309 S; 1:1000), α-Tubulin (Abcam; #ab7291; 1:5000), and GOLPH3 (Proteintech, Rosemont, IL, USA; #19112-1-AP; 1:1000). Membranes were then incubated with horseradish peroxidase (HRP)-conjugated goat anti-mouse (Merck, Darmstadt, Germany; #DC02L; 1:5000) or anti-rabbit IgG antibodies (Thermo Fisher Scientific, #31460; 1:5000) for 2 h at room temperature. Immunoreactive protein bands were detected using SuperSignal West Femto Maximum Sensitivity Substrate (Thermo Fisher Scientific; #UH289157). The quantification of western blotting was performed by Image J (Version1.8.0, NIH, USA, https:// imagej. nih. gov/ ij/ index. html) as previously described [64]. α-Tubulin, β-actin and GAPDH were used as loading controls.

### Immunohistochemistry (IHC)

Immunohistochemical analysis was used to measure GOLPH3 protein levels in colon cancer tissues. Briefly, paraffin-embedded specimens were cut into 4 μm sections and baked at 60 °C for 2 h, followed by deparaffinization with xylene, and rehydration. Sections were treated with EDTA antigenic retrieval buffer and microwaved for antigenic retrieval, treated with 3% hydrogen peroxide in methanol to quench endogenous peroxidase activity, and incubated with 1% bovine serum albumin to block nonspecific binding. Sections were then incubated with anti-GOLPH3 rabbit polyclonal antibodies (1:100, Abcam, ab69179) at 37 °C for 40 min. Normal goat serum was used as a negative control. After washing, tissue sections were incubated with biotinylated anti-rabbit secondary antibody (SP9000, Zhong Shan Jin Qiao, Beijing, China) and then with streptavidin–horseradish peroxidase complex (SP9000, Zhong Shan Jin Qiao). Finally, sections were immersed in 3.3′-diaminobenzidine, counterstained with 10% Mayer’s hematoxylin, dehydrated, and mounted.

GOLPH3 staining was independently scored by two pathologists. The proportion of positive tumor cells was scored as: 0, no positive tumor cells; 1, 1–10% positive tumor cells; 2, 11–35% positive tumor cells; 3, 36–70% positive tumor cells; and 4, >70% positive tumor cells. Staining intensity was scored as: 0, no staining; 1, weak staining (light yellow); 2, moderate staining (yellow brown); and 3, strong staining (brown). The staining index for GOLPH3 expression in colon cancer lesions was calculated by multiplying the two scores obtained for each sample, giving values of 0, 1, 2, 3, 4, 6, 9, or 12. A score ≥ 6 was defined as high GOLPH3 expression and a score ≤ 4 was defined as low GOLPH3 expression.

### Transwell assay

Cell migration was analyzed in vitro using 8.0 µm Transwell Permeable Supports (Corning Inc. Corning, NY, USA, 9335017). To evaluate cell migration, 1 × 10^4^ HCT-116-PMSCV-Vector, HCT-116-PMSCV-Vector-GOLPH3, HCT-116-pSuper-Vector, HCT-116-pSuper-GOLPH3 sh1#, and HCT-116-pSuper-GOLPH3 sh2# cells in serum-free medium (200 µL) were seeded into the upper part of a Transwell chamber. The lower chamber was supplemented with 400 µL DMEM medium containing 30% FBS, and the chamber was incubated at 37 °C for 24 h. Thereafter, the cells remaining on the upper side of the filters were removed using a cotton swab. The migrated cells on the underside of the membrane were fixed with 4% paraformaldehyde for 20 min and then stained with 0.1% crystal violet solution for 15 min. After the membrane had been washed with distilled water, the cells that had invaded the membrane were counted in three randomly chosen fields of view, and images were captured under an Olympus® CKX53 microscope (Olympus, Tokyo, Japan). A similar procedure using a Transwell insert pre-coated with 80 µL of Matrigel was performed to measure cell invasion in three independent assays.

### Wound-healing assay

HCT-116-PMSCV-Vector, HCT-116-PMSCV-Vector-GOLPH3, HCT-116-pSuper-GOLPH3, HCT-116-pSuper-GOLPH3 sh1#, and HCT-116-pSuper-GOLPH3 sh2# cells (5 × 10^6^) were seeded in a 6-well-plate and cultured in DMEM containing 10% FBS to 90% confluence. The confluent cell monolayer was wounded using a sterile 10-μL pipette tip, and the cells in suspension were washed off using a normal growth medium. Images of the monolayer wound were captured at 0, 24, and 48 h under an Olympus® CKX53 microscope, in three randomly chosen fields of view. The cell migratory ability was calculated as the ratio of the wound width after 24 and 48 h to the wound width at 0 h.

### Animal studies

The zebrafish transgenic line Tg (fli1a: EGFP) was used for the in vivo studies of the colon cancer cell lines. All animal experiments were conducted in accordance with the guidelines and approval of the respective Animal Research and Ethics Committees of Shenzhen University. Zebrafish embryos were incubated at 28.5 °C. HCT-116-PMSCV-Vector, HCT-116-PMSCV-GOLPH3, HCT-116-pSuper-Vector, and HCT-116-pSuper-GOLPH3 sh1# cells were labeled with 2 μg/mL chloromethyl (CM)-DiI (1,1’-Dioctadecyl-3,3,3’,3’-Tetramethylindocarbocyanine Perchlorate (‘DiI’; DiIC18(3))) (Thermo Fisher Scientific; Cat. NO. C7000) for 30 min at 37 °C. Before injection, zebrafish embryos at 48 h post-fertilization were dechorionated using sharp-tip forceps and anesthetized using 0.04 mg/mL tricaine. Approximately 400 tumor cells were resuspended in PBS and 20 nL of tumor cell solution was injected into the perivitelline cavity of each embryo using a micro-injector. The fish embryos were immediately transferred into E2 medium solution. Injected embryos were incubated at 28.5 °C, and the medium containing 0.45 mM propylthiouracil (PTU) was changed every day. Tumor growth and invasion were monitored using fluorescence microscopy. The number of disseminated foci from the tumor mass in the zebrafish embryo tails was counted.

### mRFP-GFP-LC3 reporter assay

After transfection with mRFP-GFP-LC3, autophagosomes were labeled in yellow (mRFP and GFP), while autolysosomes were labeled in red (mRFP only). After 36 h, the cells were fixed using 4% paraformaldehyde, and the nuclei were counterstained with DAPI (blue). The cells were then visualized using fluorescence microscopy.

### RNA sequencing

The dosing group and control group were evaluated in a 3-to-3 ratio. Cells (1 × 10^6^) were taken from three different generations of HCT-116-PMSCV-Vector and HCT-116-PMSCV-GOLPH3 cell lines, and these cell lines were lysed with 500 μL of RNAiso Plus (TaKaRa, Osaka, Japan). RNA sequencing was performed by Novogene Co. Ltd. (Tian Jin, China). The data are available in the NCBI Gene expression Omnibus database (GEO) with accession number GSE196875.

### Statistical analysis

The chi-squared test and Spearman correlation analysis were applied to explore the relationship between GOLPH3 expression and patients’ characteristics, including sex, age, tumor stage, TNM classification, and survival time. Survival curves were plotted using the Kaplan–Meier method and compared using the log-rank test. Univariate and multivariate Cox regression statistical analyses were performed to investigate the effects of patient characteristics on overall survival. All statistical analyses were performed using the SPSS statistical software package (IBM Corp., Armonk, NY, USA. In this study, a *P*-value < 0.05 was considered statistically significant.

## Supplementary information

Supplemental Figure 1

Supplemental Figure 2

Spplementary Figure Legends

uncropped original data

Author contribution

## Data Availability

The datasets used and/or analyzed during the current study are available from the corresponding author on reasonable request.
