# Supplementary figures and images for: Golgi phosphoprotein 3 induces autophagy and epithelial–mesenchymal transition to promote metastasis in colon cancer
Source: Cell Death Discov. 2022 Feb 21;8:76. doi: 10.1038/s41420-022-00864-2 (PMC8861175; doi:10.1038/s41420-022-00864-2)

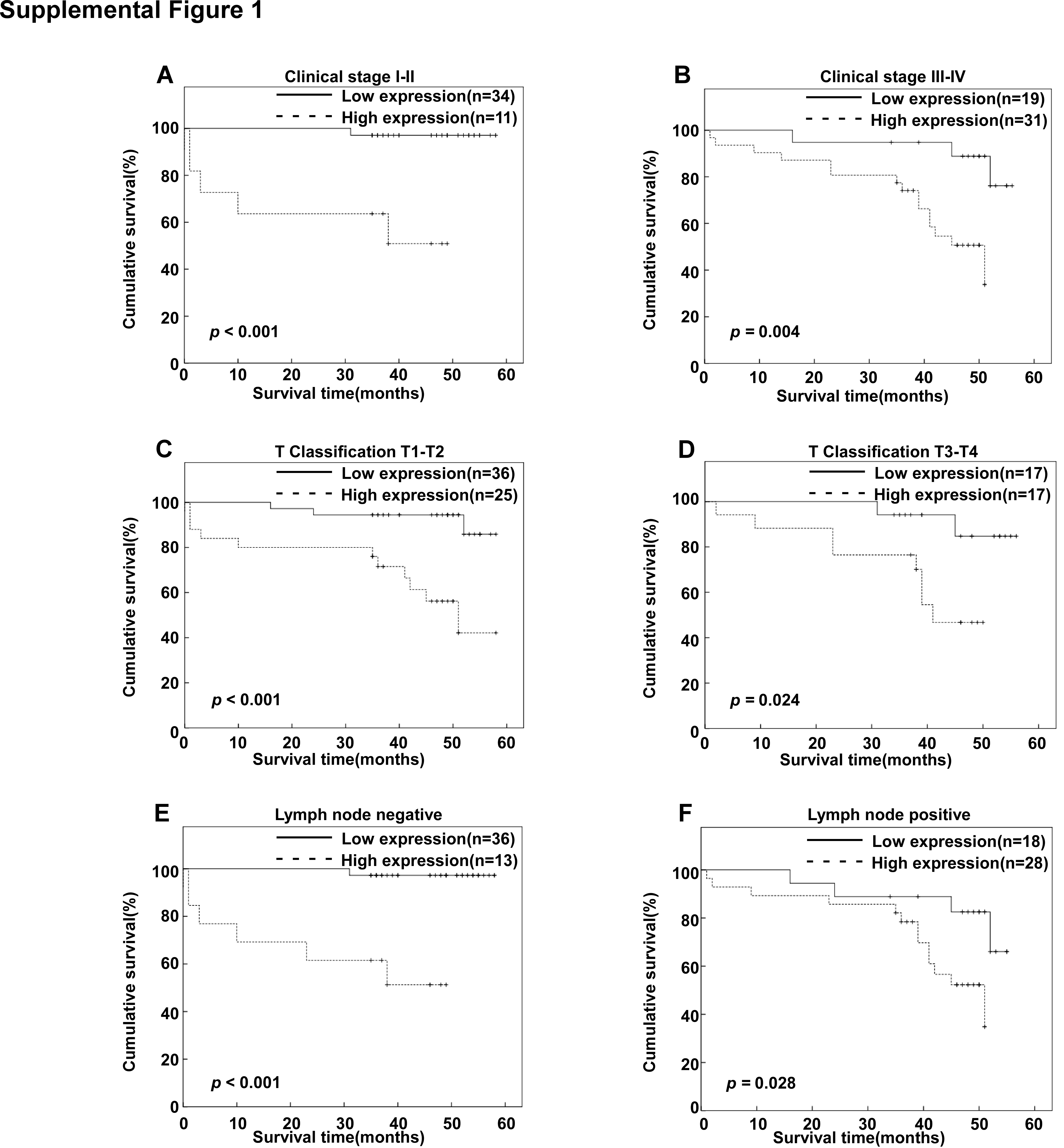

Supplement: Supplementary file 1 — Supplemental Figure 1 [file 41420_2022_864_MOESM1_ESM.tif]

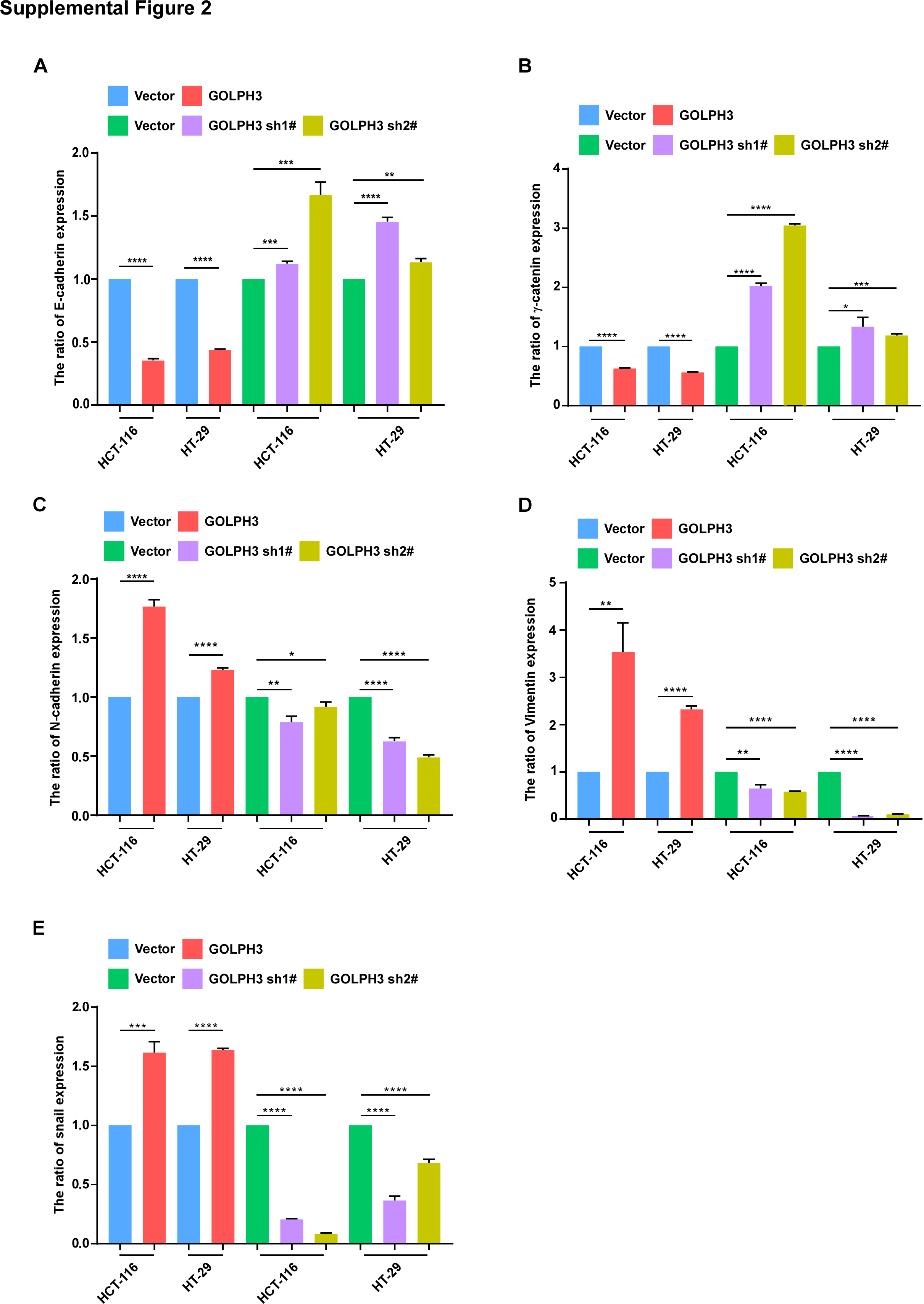

Supplement: Supplementary file 2 — Supplemental Figure 2 [file 41420_2022_864_MOESM2_ESM.tif]
