# Supplementary material for: Golgi phosphoprotein 3 induces autophagy and epithelial–mesenchymal transition to promote metastasis in colon cancer
Source: Cell Death Discov. 2022 Feb 21;8:76. doi: 10.1038/s41420-022-00864-2 (PMC8861175; doi:10.1038/s41420-022-00864-2)
Supplement: Supplementary file 3 — Spplementary Figure Legends [file 41420_2022_864_MOESM3_ESM.docx]

**Spplementary Figure Legends**

**Supplemental Fig. S1: Survival curves for various clinical subgroups with colon cancer** Kaplan–Meier curves with univariate analyses (log-rank) for patients with various clinical stages, T classifications, and lymph node metastasis statuses. *P*-values were calculated using the log-rank test. **P* < 0.05.

**Supplemental Fig. S2:** The quantification of western blotting from fig.4C-D. (**A**) The quantification of E-cadherin expression; (B) The quantification of γ-catenin expression; (C) The quantification of N-cadherin expression; (D) The quantification of Vimentin expression; (E) The quantification of snail expression.
