# Supplementary material for: Golgi phosphoprotein 3 induces autophagy and epithelial–mesenchymal transition to promote metastasis in colon cancer
Source: Cell Death Discov. 2022 Feb 21;8:76. doi: 10.1038/s41420-022-00864-2 (PMC8861175; doi:10.1038/s41420-022-00864-2)
Supplement: Supplementary file 5 — Author contribution [file 41420_2022_864_MOESM5_ESM.pdf]

DECLARATION OF CONTRIBUTIONS TO ARTICLE

ADMC

Manuscript Number: CDDISCOVERY-21-2993R

Journal Name: *Cell Death Discovery* (the 'Journal')

Proposed Title of the Contribution:

Golgi phosphoprotein 3 induces autophagy and epithelial–mesenchymal transition to promote metastasis in colon cancer (the 'Contribution)

Author(s):

Li-Yun Gong, Ting Tu, Jing Zhu, Ao-Ping Hu, Jun-Wei Song, Jing-Qiang Huang, Yi Yang, Zeyao Zhu and Yu Chen (the 'Authors')

For all *CDDis* articles, each person named as an author in the published version must be able to show he or she has contributed substantially to the article.

Authorship credit should be based on 1) substantial contributions to conception and design, acquisition of data, or analysis and interpretation of data; 2) drafting the article or revising it critically for important intellectual content; and 3) final approval of the version to be published. Authors should meet conditions 1, 2 and 3.

Any person who cannot be shown to have made a substantial contribution to the article cannot be listed as an author in the final version. The name of any person who is deemed to have made a minor contribution can, however, appear in the Acknowledgments section of the article.

Please complete the table below to indicate the contributions of all named authors to the manuscript.

Author Full Name:	Specification of Contribution to the Manuscript:
Li-Yun Gong	Performed the experiments, wrote the paper and supervised this study
Ting Tu	Performed the experiments and analyzed data
Jing Zhu	Analyzed data and help conduct partial experiment
Ao-Ping Hu	Analyzed data and help conduct partial experiment
Jun-Wei Song	Analyzed data and help conduct partial experiment
Jing-Qiang Huang	Analyzed data and help conduct partial experiment
Yi Yang	Provided guidance on experimental technology and gave suggestions
Zeyao Zhu	Provided guidance on experimental technology and gave suggestions
Yu Chen	Writing, review, revision of the manuscript

Please complete the table below to indicate the contributions of all named authors to the figures.

Figure 1:

Li-Yun Gong and Jing Zhu performed IHC data analysis. Ting Tu performed Western blot assay. Yu Chen provided guidance on experimental technology and gave suggestions.

Figure 2:

Li-Yun Gong and Jing Zhu constructed GOLPH3 stable cell lines. Li-Yun Gong and Jing Zhu performed transwell assay and transwell matrix invasion assay and Wound Healing assay. Li-Yun Gong and Yi Yang provided guidance on experimental technology and gave suggestions.

Figure 3:

Ting Tu and Jing Zhu performed Zebrafish embryos models. Li-Yun Gong and Zeyao Zhu analyzed data.

Figure 4:

Li-Yun Gong and Ting Tu performed EMT western-blot, IHC and HE staining. Li-Yun Gong and Jun-Wei Song analyzed sequencing data.

Figure 5:

Li-Yun Gong and Jun-Wei Song performed GSEA analysis. Jing Zhu performed immunofluorescence staining assay. Ting Tu and Jing-Qiang Huang performed western blots. Li-Yun Gong and Yu Chen analyzed data.

Figure 6:

Li-Yun Gong and Ting Tu performed western-blot. Jing Zhu and Ting Tu performed Zebrafish embryos models. Li-Yun Gong and Zeyao Zhu analyzed data.

Signed for and on behalf of the Author(s): LiyunGong

Print Name: Li-Yun Gong

Date: 2022-01-14

Please complete the table below to indicate the contributions of all named authors to the figures.

Figure 7:

Jun-Wei Song performed GO analysis. Li-Yun Gong and Ao-Ping Hu performed WB. Li-Yun Gong, Yi Yang, Yu Chen and Zeyao Zhu analyzed data. Jing-Qiang Huang designed model.

Supplementary Figure1:

Li-Yun Gong and Jing Zhu performed IHC data- survival curve analysis.

Supplementary Figure2:

Ting Tu performed the quantification of western blotting from fig.4C-D.

Signed for and on behalf of the Author(s):

Print Name: Li-Yun Gong

Date: 2022-01-14
